# Book Reviews

**Published:** 1807-02

**Authors:** 


### CRITICAL ANALYSIS

OF THE

### RECENT PUBLICATIONS

ON THE

### DIFFERENT BRANCHES OF PHYSIC, SURGERY, AND MEDICAL PHILOSOPHY.

---

*The Edinburgh Medical and Surgical Journal,* Number No. IX. for January, 1807.

---

Article 1.—*Case of Extirpation of the Eye.* By Mr. W. Mudd, Surgeon, Hadleigh.

Though this operation proved ultimately unsuccessful, yet the minuteness with which it is related, does great credit to the author. If it is deficient in any respect, it seems to be in the describing the contents of the tumour. Even this may be readily excused by the violence the parts had suffered, and the changes which had taken place before Mr. Mudd was consulted. By the description, after excision, we suspect the tumour must have consisted of that species of cancer which Dr. Adams calls *hydatis cruenta*; not a vestige, says our author, of the organization of the the eye remained; it was a confused mass, considerably vascular, of a livid colour, and in consistence resembling, but more spongy than that of liver.

Article 2.—*Cases of Preternatural Pulsation in the Abdomen.* By J. A. Albers, Physician in Bremen.

This disease, or rather symptom, has been very accurately remarked by Morgagni, and by our English Physician Dr. Whitt. It is a very common attendant on Hysteria, and still more on Hypochondria. Of late years it has been too much overlooked by those systematic writers who have undertaken to comprise every thing in a certain number of volumes. The cases here related are well worth perusing, and show much attention and candour in the compiler. Some he imputes to accumulated fæces, others to tumours of different kinds, and some to real aneurisms. In some he could discover no apparent cause; these were usually in hysterical hypochondrical subjects. The author imputes them to *diseased nerves* in the abdomen. That they arise from some of those causes which we usually call nervous, cannot be questioned; but from their irregularity, and the space they occupy, we have always considered them as the immediate effect of the subsultus of some muscle in the region of the parts affected.

Article 3.—*Observations on Hydrocephalus Internus.* By G. G. Kuhn, Professor of Medicine, and Rector Magnificus of the University of Leipsick.

This is an useful paper, in calling the attention of practitioners to the various causes of this formidable disease. We are by no means satisfied with the few causes assigned by the author, but the hints are very useful, as it must be confessed that the English, to whom the Professor gives the credit of first marking the disease, have been too inattentive to enquiring into many minute particulars which might have directed their treatment.

Article 4.—*History of an Injury to a Nervous Filament of the Fore Arm.* By R. J. B. Vervinet, M. D. of the School of Paris.

Article 5.—*Case of sudden Death occasioned by the Inspiration of Nitrous Gas.* By J. B. Desgranges, M. D. Member of the College of Surgeons, at Lyons.

These two papers are from French Journals, and selected with more judgement than some others we have noticed in our former remarks. The first is a valuable case from the success with which it was treated. A clean wound, in the fore arm, was attended with more than usual pain, and though readily healed, yet, after cicatrization, the pain continued, attended with spasms, stinging pains, and even convulsions; the voluntary motions of the wrist and fingers being disturbed and impeded. After a variety of remedies, the patient was relieved by the actual cautery. The author thor imputes the injury to a wound of a branch of the musculo-cutaneous nerve. Whether such was the case or not we pretend not to determine, but we think the suggestion of the actual cautery very judicious, as a means of preventing the speedy reunion of the divided parts, by which the communication of irritation was prevented also.

The other paper is not less important. It contains an account of a man and a dog who were both killed by inhaling nitrous gas, not immediately, but after a succession of symptoms, which seem to prove, that several important organs were gradually, perhaps chemically, injured. It does not appear that either was opened. Should a similar event occur, we cannot help suggesting the propriety of exhibiting, almost incessantly, water, weakly impregnated with any kind of alkali, and also the vapours of volatile alkali.

Article 6.—*Observations on the Effect of Nitric Acid in Elephantiasis.* By Edward Cook, Assistant Surgeon, Sythet; in a Letter to Francis Balfour, Esq. President, and the Members of the Medical Board, Calcutta.

We lament, that histories so crude, should be offered of diseases and remedies so important. Many of the cases related by Mr. Cook, are described in such general terms, that it is not easy to discover more, than ill conditioned and ill treated ulcers. It is not difficult to cure such by the commonest remedies, if the patient is strictly attended to, instead of trusting to his own negligence, or even to his own wish to excite compassion, as appears to have been the case in some of Mr. Cook's cases.

The paper from the Asiatic Researches follows, giving an account of " the cure of Elephantiasis and other impurities of the blood by Arsenic." This is liable to the same objection, for we are neither informed what is meant by elephantiasis, nor by other impurities in the blood. There is even some obscurity in the manner of preparing the remedy.

Article 7.—*Account of an Indian Recipe for the Tape Worm.* By Francis Buchanan, M. D. Surgeon to the Bengal Establishment.

The most important ingredient in this remedy is pomegranate root. The author of the paper remarks, " Mr. Russel, Mr. Shoolbred, and I, have given the medicine in several cases, and in none has it failed." It appears as if the tape-worm of the East Indies were different from the European.

Article 8.—*Case of Stricture of the Urethra, followed by extensive Gangrene of the Scrotum and Perinæum.* By Mr. C. Anderson, Fellow of the Royal Collogo of Surgeons, Edinburgh.

Article

Article 9.—*On the Application of Galvanism in the Cure of Diseases.* By Anthony Mongiardini, M. D. Member of the Italian National Institute.

This paper confirms what most practitioners have at last reluctantly admitted, viz. that the effects of Electricity and Galvanism are too uncertain to be depended on, though it may be right to try them in cases which resist other remedies. Among the established laws of Galvanism, we meet with a position contradictory to the observations of our English Physician, who has attended so closely to the subject.

"Galvanism (says Dr. M.) disposes bodies to putrefaction. Muscular fibres, blood, urine, and bile, when galvanized, became putrid sooner than the same substances not galvanized. I must confess my surprise, that the opposite has been maintained by the celebrated Dr. Fowler, who, speaking of the differences between electricity and galvanism, has said, that the former disposes bodies to corrupt, while the latter preserves them. I cannot quote a great number of experiments in opposition to this philosopher, but those I have made were exact and precise. Perhaps, the longer or shorter application of galvanism to the above mentioned substances, may have contributed to vary the effects. But I shall examine this subject on another occasion."

It is most probable that both these gentlemen are right. If the Galvanic shock is applied with such force, or frequency, as to kill the part quickly, putrefaction takes place earlier; but if in a slighter degree and not repeated, death will be more gradual, the muscular parts will contract or stiffen, and putrefaction will be delayed.

Article 10.—*Report of the Medical Faculty of Keil, to the Royal German Chancery of Copenhagen, relative to the Cow-Pox, in the Dutchies of Schleswig and Holstein.*

This report contains many judicious remarks, but few of them new to the English. We are glad, however, to see the candour of our continental brethren, who acknowledge, that inoculation of Cow-Pox from one human subject to another, was unknown till Dr. Jenner's publication, and that even Vaccination from the cow, when intentional, was for the most part a secret practice.

The origin of the disease from the horse is much questioned.

Article 11.—*Case of Femoral Aneurism, reaching as high as Poupart's Ligament, cured by tying the External Iliac Artery.* By John Abernethy, F. R. S. &c.

It is with the highest satisfaction that we announce this truly valuable case, which does so much honour to the courage, skill, and operative talents of Mr. Abernethy. In order to give as much publicity as possible to so meritorious an operation, we shall transcribe the whole in the words of the author.

"Jane

"Jane Field, aged 40, who had been in the habit of drinking to excess, was admitted into St. Bartholemew's Hospital, with a very large femoral aneurism, reaching as high as Poupart's ligament. The whole limb was œdematous, but in no very considerable degree. She was quite incapable of using the least exercise, or of sitting upright; and, even in bed, she suffered continual pain, which was much aggravated during the pulsation of the aneurism. The pain was so violent as to preclude sleep. She had no appetite; her pulse was feeble and frequent, generally exceeding 100; but her tongue was not furred; and her bowels were regular.

"On Saturday, October 11, the operation was performed in the same manner as in the last case. An incision, about three inches in length, was made through the integuments of the abdomen, in the direction of the artery, beginning just above Poupart's ligament. Having divided the skin and aponeurosis of the external oblique muscle, I introduced my finger between the margin of the internal oblique and transverse muscles and the peritonæum. I then divided their lower edges upwards, in the direction of the external wound, to the extent of an inch and a half, with a probe-pointed bistoury. Having thus made room for the admission of my finger, I put it down upon the artery, felt its pulsations, and gently insinuated it beneath the vessel; and then, with the aneurismal needle, passed under it two thick ligatures, carrying them upwards and downwards, as far as the detachment of the artery permitted, and tying them as firmly as I could. I next divided the artery in the interspace, but much nearer to the lower ligature than to the upper one. The wound was afterwards closed, in the middle by a ligature, and in other parts by sticking plaster. Upon removing the patient to bed, she complained of great pain in the wound, and in her head; and was very restless and ungovernable. She wished for something to procure sleep, and I gave her twenty-five drops of laudanum. This, instead of having the desired effect, made her much more restless; she was continually changing her position in bed, and complaining of violent head-ach. At night she became more tranquil. The one foot was much colder than the other; but the limbs at the knees were nearly of an equal temperature.

"Sunday, 12th, I visited her early in the morning, and found that she had been moderately quiet during the night; that she had suffered much pain in her foot, but none in the wound. The pain in the limb she described as having first attacked the thigh, next the leg, and afterwards the foot, which last pain had now ceased. The foot was warmer than it was the preceding evening, and in a state of perspiration: it was four degrees of heat lower, by Fahrenheit's scale, than that of the healthy limb. The superficial veins of the leg were filled with blood. Her pulse was 96. She had no appetite. I left her with a promise to visit her again at night, recommending her to lie quiet, and take some simple nourishment.

About

About noon, one of the dressers, observing that her skin was hot, and the tongue dry, gave her some saline medicine, with a small quantity of antimonial wine, which occasioned vomiting, and such continued nausea, that she refused all kind of food. The limb, at night, continued in the same state as in the morning. She was free from pain; her pulse 120. As she had had no evacuation, I gave her a pill, containing two grains and a half of pil. aloet. e myrrh. with the same quantity of colocynth, ordering it to be repeated in the morning, if necessary.

"Monday, 13th, The foot was nearly of the same temperature with the other. She had two stools and felt much more comfortable. Still, however, she had an aversion to all kinds of nourishment. Her pulse was 150 and 160, at different times of the day. I may here mention, that every subsequent day, she had one or more stools, without having recourse to opening medicine; and whenever she was more irritable or disturbed than usual, she had a tendency to purging. In the evening of this day, I inquired if she had a wish for any particular kind of nourishment; and, at her suggestion, gave her half a pint of porter, with some ginger and toasted bread. This seemed to agree with her stomach, as she slept the whole night, and awoke much refreshed the next morning. Her tongue was then clean; she took some tea and muffin for breakfast, and broth and bread in moderate quantities, in the course of the day. Half a pint of porter was allowed her at dinner and supper. Her pulse this day, (Tuesday) was 95. The foot warmer than the other. The wound was dressed for the first time; it appeared well closed, and discharged but little. Wednesday, Pulse about the same number; had slept during the night, but not so soundly as on the preceding one. The wound and contiguous parts were tender; there was a considerable discharge, which was fetid; the lower ligature came off the artery. The artery, as I have mentioned, was divided very near to the lower ligature; and it is probable, that, in the restlessness of the patient, subsequent to the operation, the motions of the limb had drawn the artery from out of the ligature.

"I have never made use of the expedient suggested by Mr. Henry Cline, for securing ligatures upon arteries, since I never felt its necessity; and because I have always thought it right to tie a large artery with so thick a ligature, that it would have been unsuitable to the practice which he has recommended. One advantage arising from tying a large artery with a thick ligature is, that it may be drawn as tight as possible, without apprehension of cutting the vessel, or of its speedily coming off from it. Should I, in any future instance, think it right to oppose any mechanical obstacle to the ligature's coming off the vessel which it encircles, I should do it in the following manner, which, by way of suggestion, I take the liberty of mentioning. Having tied a large knot at one end of a small thread, I would pass it, by means of a common sewing needle, through the middle of the artery, in front of the ligature ligature which encircles it; I would then form a second thick knot on the thread, close upon the surface of the vessel. These two knots, would, I think, present a considerable obstacle to the slipping of the circular ligature from off the end of the artery."

If there is any thing in which our northern neighbours have the advantage over us, it is in the habit of reading more than ourselves. Whether the quantity of interesting events which our numerous hospitals afford, and the travelling from the one end to the other of this overgrown metropolis, engross our whole thoughts as well as time; but certain it is, that excepting on a work, or a subject which becomes matter of general conversation, our practitioners, who are most employed, seem perfectly ignorant of the labours of others. We were led to these suggestions by the note just quoted. It is impossible not to regret, that the labours of Mr. Jones should be so entirely overlooked, that Mr. Abernethy, should conceive he owes any thing to his thick ligatures. Does he conceive himself endowed with sufficient muscular force to cut through a living iliac artery, by any ligature ever used in surgery. When Mr. Astley Cooper published his adoption of Mr. H. Cline's plan, those valuable experiments of Dr. Jones * were not before the public. We cannot help wishing Mr. Abernethy's patient perusal of that work, and earnestly entreating him, or some other anatomical teacher, to complete the few desiderata that are wanting in so important an enquiry.

Article 12.—*Observations on the Epidemic Ophthalmia, as it affected the Troops, in Hyth Barracks.* In a Letter to George Peach, Esq. Surgeon of the Second Battalion, 52d Foot, at Reading-street Barracks, Kent, to James M'Gregor, Esq. M D. Deputy Inspector-General of Hospitals, Portsmouth.

The principal diagnostic symptoms of this disease, are the suddenness and severity of the attack, the tendency to inflammation of the globe of the eye, the almost uniform first sensation of sand rolling in the eye. The method of cure was not less uniform, consisting in bleeding, ad deliquium, repeating the operation as often as was necessary. If this remedy was adopted early, it rarely failed of success. If the disease became afterwards chronic, it was treated according to the symptoms.

Article 13.—*Observations on the Effect of evacuating the aqueous Humour in the Inflammation of the Eyes; and the Changes produced in the Transparency of the Cornea, from the Increase or Diminution of the Contents of the Eye-Ball.* By James Wardrop, Fellow of the Royal College of Surgeons, in Edinburgh.

It appears by some experiments of Dr. Barclay, that pressure on the cornea lessens the transparency of that substance; this was

* See Dr. Jones's Treatise on Hæmorrhage, in our Analysis of Books Vol. 15 and 16.

 proved proved in a variety of instances in the eyes of dead animals. The facility with which the cornea heals after puncture, in many operations, and the little uneasiness felt by the patient, induced Mr. Wardrop to try the effect of evacuating the aqueous humour in those cases, in which the cornea appeared opake, and the volume of the eye large and projecting. This will be best understood by transcribing one of his cases.

"A gentleman, about twenty-one years of age, had a very violent inflammation of the left eye-ball. The *sclerotica* was covered with numerous scarlet-coloured blood-vessels; but none of them passed over the transparent cornea. The anterior chamber was turbid; and several small spots, of a matter resembling pus, were seen in it, towards the circumference of the cornea. The pupil was much contracted, the eye-lids swelled, and their external surface covered with varicose veins. There was a constant flow of acrid tears. Vision was almost entirely destroyed; but, notwithstanding, the eye was extremely sensible to light. He had great pain in the eye-ball, and constant headach, with a sense of fulness in the orbit. The inflammation had begun five weeks before, without any known cause, and every symptom had since gradually increased. I made an incision with an extracting-knife through the cornea, and the aqueous humour spirted out. From the difficulty in securing the eye-ball, the pressure employed gave considerable pain. The incision smarted for two or three minutes; but, before I left the room, he said he found great relief, and that the pain in the eye-ball and head, and the peculiar feeling of weight and distension were entirely removed. On examining the anterior chamber, all the turbidness had disappeared, and the cornea seemed perfectly transparent. He was advised to do nothing but foment the eye and the neighbouring parts, and to take a purge. As he lived at some distance from town, I had no opportunity of visiting him afterwards; but I was informed by Dr. Mitchell of this place, with whom I had seen him, that an hour after the operation, all the pain, occasioned by the incision and the pressure, was entirely gone, and the eye had become quite easy. In two days all redness went off, and Dr. Mitchell, who saw him five days afterwards, said, that he could not discover the mark of the incision; that the inflammation had disappeared; and that his vision was nearly as perfect as it had been before the commencement of the disease. Some months afterwards, I heard that he continued perfectly well."

Every addition to our remedies in so important and delicate an organ, is highly deserving attention, especially when unattended with danger, as the present operation seems to be.

These are all the original articles. Among the medical intelligence we meet with the following letter to Dr. Harrison, which we transcribe with great pleasure, and sincerely wish the same candour candour had been observed in all the other communications on this subject.

"*Copy of a Letter, on the Subject of Medical Reform, sent to Dr. Harrison, of Horncastle, Lincolnshire.*"

"SIR,

"I am directed, by the Royal College and Corporation of Surgeons of Edinburgh, to inform you, that they have perused with attention the Plan of Medical Reform, contained in the printed copy of correspondence with which you have been pleased to favour them.

"From the most correct information which they have been able to procure, the College are inclined to believe, that the practice of medicine in this country, so far from being in a low and degraded state, is, at present, in all its branches, on a more respectable footing, and its practitioners better educated, than in any former period. The College have had much satisfaction in observing, for a series of years, the gradually increasing knowledge and acquirements of those who present themselves to the College as candidates for surgical diplomas.

"The College, however, are by no means disposed to deny that abuses exist in the practice of medicine, in this as well as in other countries; but they are, at the same time, convinced, that the powers and privileges with which the different Universities, Colleges, and Corporations of Physic and Surgery, in this part of Great Britain, are already invested, are sufficient, if duly exercised, for the correction of the greater part of these abuses; while other abuses, and those of which your correspondents chiefly complain, are not of a nature to be easily rectified by legislative interference, or by legal prosecutions.

"With this conviction deeply impressed upon their minds, you will readily perceive that it is unnecessary for the College to enter into any discussion of the merits of the Plan of Reform which the state of medical practice in this country does not seem to them to require; and which does not provide better for the instruction of future candidates for surgical diplomas than the regulations already adopted by the College, a copy of which I have been directed to transmit to you along with this letter.

"I have the honour to be, Sir, your most obedient humble servant,

"WILLIAM FARQUHARSON,

"President of the Royal College and Incorporation of Surgeons of Edinburgh."

That empiricism exists cannot be doubted, but as far as our own knowledge extends, the empiricism of mere remedies is much lessened, and the trade is gradually transferred to the privileged orders. Mr. Martin Bree and Mr. Taylor, call themselves Members of the Corporation of Surgeons. These gentlemen advertise in a regular way; others more indirectly, with all the trick of the puff oblique, the puff reversed, the puff collusive, or the puff by inference: In short, the various shades of empiricism are so numerous, that we fear the requiring all practitioners to procure licences to kill, would only render quackery more impudent by being more secure.

